# Supplementary material for: Crystallization Behavior and Physical Properties of Monoglycerides-Based Oleogels as Function of Oleogelator Concentration
Source: Foods. 2023 Jan 11;12(2):345. doi: 10.3390/foods12020345 (PMC9857595; doi:10.3390/foods12020345)
Supplement: Supplementary file 1 [file foods-12-00345-s001.zip › foods-2099057-supplementary.pdf]

foods MDPI

Supplementary materials

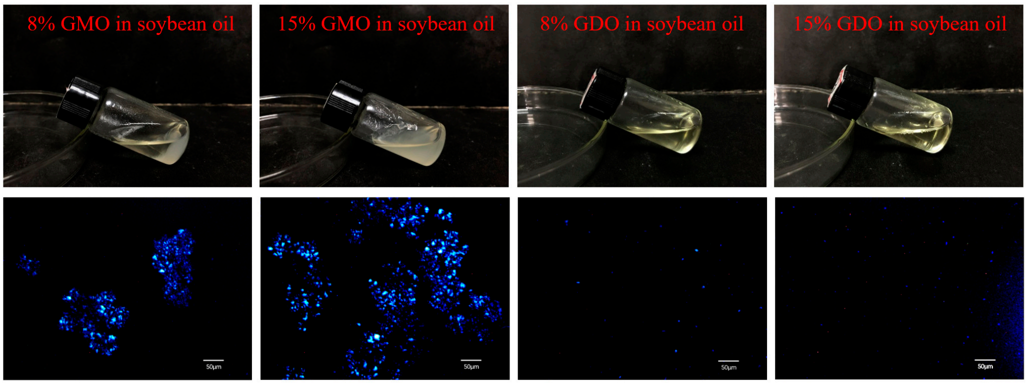

Figure S1. Visual appearance and polarized light microscopy images of oleogels prepared by monoolein (GMO) and diolein (GDO). The scale bar is 50 μm.

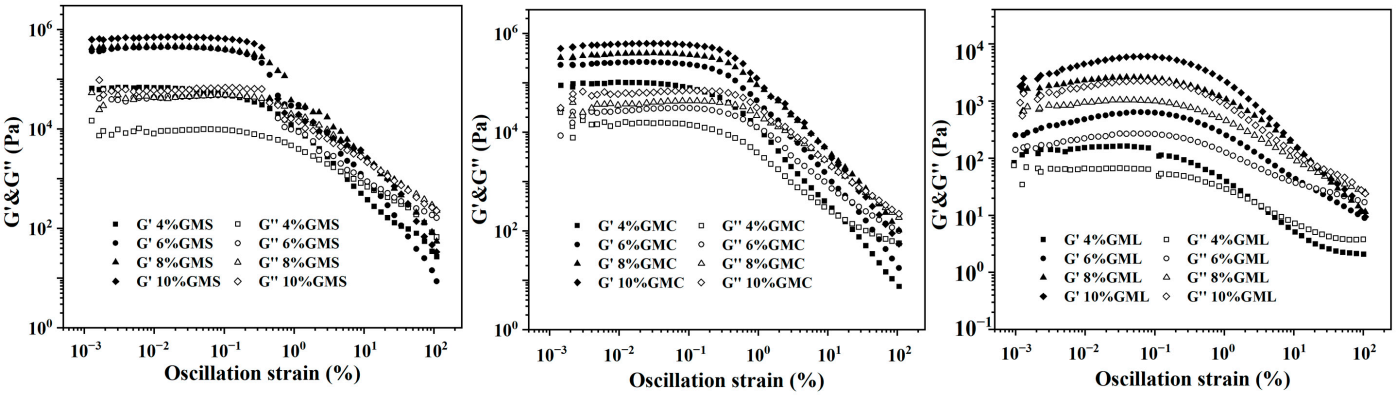

Figure S2. Strain sweep curves of oleogels prepared by GMS, GMC and GML with different concentrations.

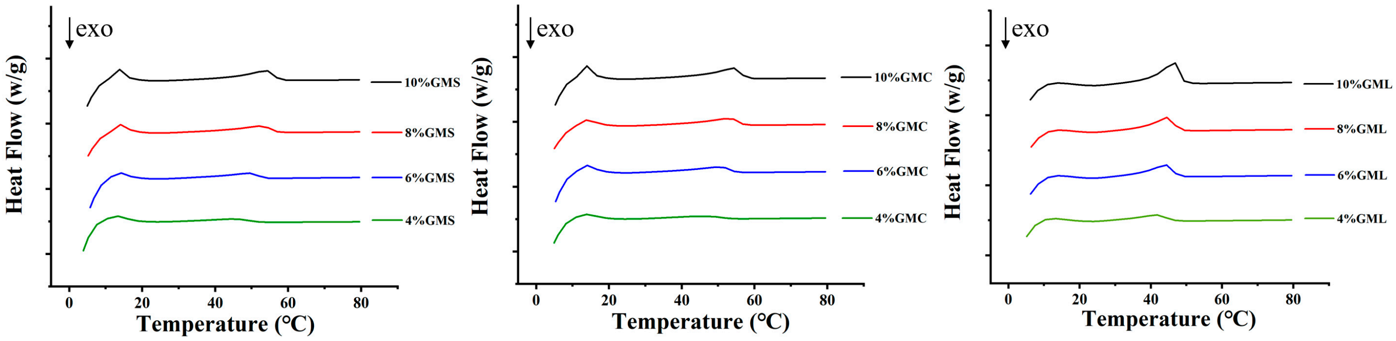

Figure S3. DSC patterns of oleogels prepared by GMS, GMC and GML with different concentrations.

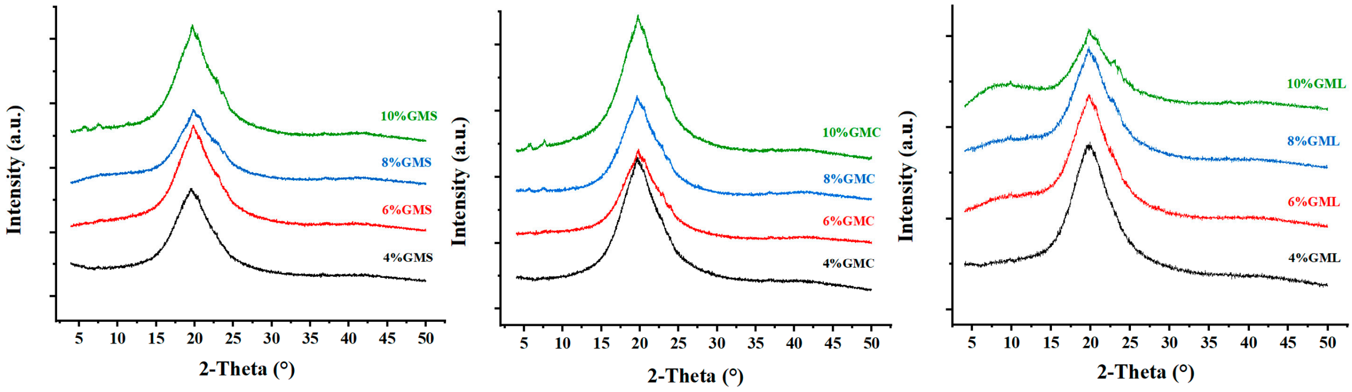

Figure S4. The X-ray Diffraction Patterns of the oleogels.

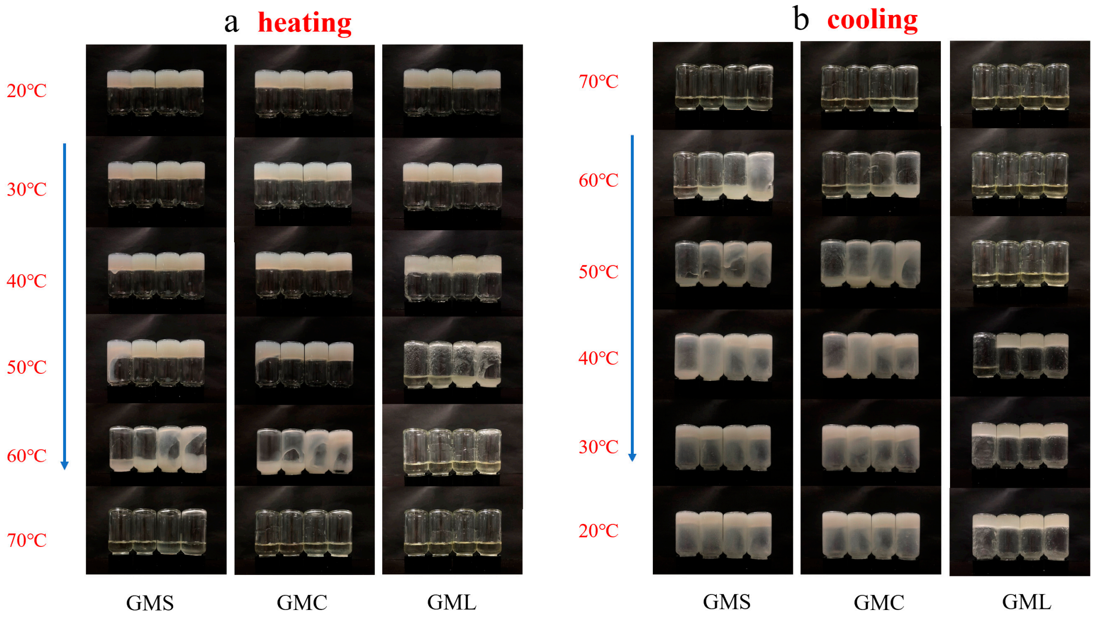

Figure S5. Visual appearance of oleogels during heating (a) and cooling process (b). The concentration of MAG from left to right is 4 wt%, 6 wt%, 8 wt%, 10 wt%.

Table S1. DSC melting parameters, onset temperature (Ton), peak temperature (Tp), end temperature (Tend) and change in total enthalpy (△H) for oleogels.

Sample	T_{on} (℃)	T_p (℃)	T_{end} (℃)	△H (W/g)
4%GMS	40.85±0.15^a	46.95±2.25^a	52.50±2.10^a	3.04±0.06^a
6%GMS	42.40±0.10^b	49.10±0.30^{ab}	53.70±0.50^{ab}	5.26±0.04^b
8%GMS	45.30±0.00^c	53.40±1.00^b	58.35±1.25^b	7.39±0.08^c
10%GMS	46.05±0.15^d	54.00±0.20^b	58.00±0.10^b	9.04±0.08^d
4%GMC	43.25±0.55^a	49.25±3.45^a	54.05±1.25^a	2.93±0.02^a
6%GMC	43.70±0.30^a	50.05±0.05^a	54.50±0.10^a	5.18±0.08^b
8%GMC	46.10±0.60^b	53.45±0.55^a	57.80±0.80^b	7.54±0.04^c
10%GMC	46.30±0.10^b	54.10±0.10^a	58.40±0.40^b	9.06±0.04^d
4%GML	34.65±0.25^a	41.30±0.50^a	46.25±0.25^a	2.16±0.08^a
6%GML	36.70±0.70^{ab}	44.35±0.15^b	47.70±0.10^b	3.85±0.24^b
8%GML	38.35±0.95^{bc}	45.00±0.30^{bc}	47.95±0.05^b	5.05±0.23^c
10%GML	40.50±0.20^c	46.10±0.20^c	48.55±0.55^b	6.43±0.16^d

Different lower-case letters (a, b, c, d) mean significant differences ($p < 0.05$) between different concentration (the same MAG).
